# Characterization of high-artemisinin yielding *Artemisia annua* bioecotypes using gene-specific STS markers and HPLC for quality-oriented selection

**DOI:** 10.1016/j.bbrep.2026.102637

**Published:** 2026-05-23

**Authors:** Mohammad Taher Hallajian, Sahar Tavakoli Mohammadi, Mohammad Ali Ebrahimi, Mohammad Reza Naghavi, Mojtaba Kordrostami

**Affiliations:** a Nuclear Agriculture Research School, Nuclear Science and Technology Research Institute (NSTRI), Karaj, Iran; b Department of Agricultural Biotechnology, Faculty of Agriculture, Payam Noor University Alborz Branch, Karaj, Iran; c Division of Biotechnology, Department of Agronomy and Plant Breeding, College of Agriculture and Natural Resources, University of Tehran, Karaj, Iran

**Keywords:** *Artemisia annua*, Artemisinin, STS marker, Artemisinin biosynthesis, Molecular diversity, Marker-assisted selection (MAS), HPLC, PCA, Secondary metabolites

## Abstract

*Artemisia annua* is the sole natural source of artemisinin, an essential compound for malaria treatment. However, variable yields among bioecotypes hinder consistent production. This study aimed to characterize 17 *A. annua* biotypes using gene-specific STS markers targeting key enzymes in the artemisinin biosynthesis pathway and to evaluate their association with artemisinin content for quality-oriented selection of plant material. Eleven STS primer pairs were designed for genes including *ADS*, *CYP71AV1*, *DBR2*, *ALDH1*, *HMGR*, *TTG1*, and others. PCR amplification, gel electrophoresis, and HPLC quantification of artemisinin were performed. Molecular marker efficiency indices were calculated. Hierarchical clustering, PCA, correlation heatmaps, and radar chart visualizations were employed to assess genotype–metabolite relationships. A total of 10 markers were polymorphic across biotypes, with significant correlation to artemisinin content (R^2^ > 0.85 for key markers). Cluster and PCA analyses grouped biotypes into high-, medium-, and low-producing clusters. Biotypes 260 and 316 consistently displayed superior marker profiles and artemisinin content (>2.7 mg/g DW). Marker indices such as PIC, MI, and EMR highlighted *TTG1*, *DXR*, and *ADS* as the most informative loci. Radar chart analysis confirmed these markers and genotypes as optimal targets for selection. Gene-targeted STS markers can effectively distinguish elite *A. annua* biotypes with high artemisinin content. This integrated molecular-metabolite framework supports marker-assisted breeding and transgenic improvement with relevance to food, nutraceutical, and functional ingredient applications.

## Highlights

• STS markers for artemisinin biosynthesis were applied to 17 *A. annua* biotypes.
• Marker presence strongly correlated with artemisinin levels (R^2^ > 0.85).
• Biotypes 260 and 316 had high marker density and >2.7 mg/g DW artemisinin.
• PCA and clustering confirmed marker-metabolite links and genotype diversity.
• Results support marker-assisted breeding of high-artemisinin *A. annua* lines.

## Graphical abstract

**Figure:**
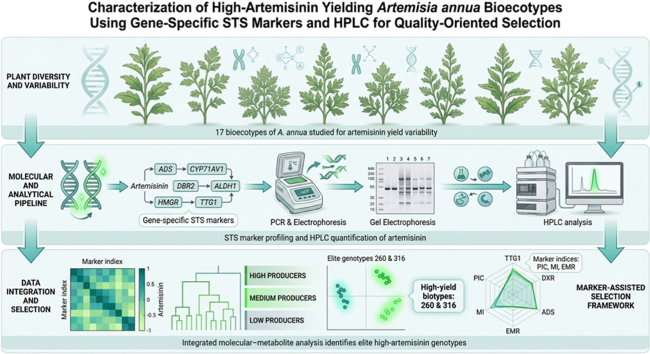

## 1 Introduction

Medicinal plants have long been a cornerstone of both traditional and modern healthcare systems, offering a vast array of bioactive compounds essential to pharmaceutical innovation [1,2]. Among these, *Artemisia annua* L., commonly known as sweet wormwood, holds a special place due to its role in producing artemisinin—a sesquiterpene lactone with a unique endoperoxide bridge [3]. Artemisinin is widely recognized as the most effective treatment for malaria caused by *Plasmodium falciparum* [4]. Since its discovery in China during the 1970s and the subsequent awarding of the Nobel Prize in Physiology or Medicine in 2015, artemisinin and its derivatives (such as artesunate, artemether, and dihydroartemisinin) have become the cornerstone of artemisinin-based combination therapies (ACTs), which are currently endorsed by the World Health Organization as the frontline treatment against drug-resistant malaria [5]. Despite its proven efficacy, the commercial supply of artemisinin faces persistent challenges [6]. Natural production in the plant is low—often less than 0.5% of dry weight—and varies significantly between genotypes [7]. Additionally, chemical synthesis remains inefficient and costly. These constraints have spurred worldwide interest in strategies to improve artemisinin yields, including metabolic engineering, tissue culture, and—critically—the identification of high-producing natural genotypes [8].

Artemisinin biosynthesis is governed by a complex enzymatic cascade beginning with amorpha-4,11-diene synthase (*ADS*) [9] and proceeding through several redox and cyclization steps involving key enzymes like *CYP71AV1*, *DBR2*, *ALDH1*, *HMGR*, and *FPS* [10]. The regulation and expression of these genes are central to the plant's capacity to synthesize artemisinin [2]. Therefore, molecular profiling of *A. annua* germplasm using gene-specific markers provides a powerful and targeted approach for identifying elite genotypes with superior biosynthetic potential [11].

Among available molecular tools, Sequence Tagged Site (STS) markers are especially useful for such applications [12]. These short, gene-targeted DNA fragments are highly specific, reproducible, and capable of detecting functional allelic variations [13]. When designed from genes involved in artemisinin biosynthesis, STS markers can be used as predictive tools to screen *A. annua* populations for high-yield potential. This molecular diagnostic approach is particularly valuable for countries like Iran, where the local germplasm remains genetically undercharacterized despite harboring a wealth of natural *A. annua* ecotypes adapted to diverse climates.

While earlier studies on *Artemisia annua* genetic diversity have predominantly relied on anonymous marker systems such as RAPD [14], ISSR [15], and AFLP [16], these approaches primarily capture neutral genomic variation without direct linkage to biosynthetic function. In contrast, sequence-tagged site (STS) markers designed from known genes of the artemisinin biosynthetic pathway provide a function-oriented alternative that directly reflects the genetic architecture underlying metabolite accumulation. Gene-specific STS markers enable the detection of allelic presence, absence, or structural variation within key enzymatic loci [17] such as *ADS*, *CYP71AV1*, *DBR2*, and *ALDH1*, thereby offering greater biological interpretability for marker-assisted selection. This targeted strategy allows breeders to prioritize genotypes based not only on genetic diversity but also on pathway completeness and biosynthetic potential, representing a practical advancement over anonymous marker systems for artemisinin-oriented breeding programs.

The novelty of this study lies in its integrated approach—merging gene-targeted STS marker analysis with phytochemical quantification via HPLC—to perform the first molecular and metabolite-level characterization of Iranian *A. annua* biotypes. While previous studies have largely focused on morphological traits or general genetic variability, our research zeroes in on key genes in the artemisinin pathway and directly correlates their amplification with actual artemisinin levels. This dual-layered methodology enables a more precise and functional selection of elite genotypes.

Among the 17 biotypes screened, two genotypes—biotype 260 and biotype 316—stood out for their combined high expression of multiple biosynthetic genes and elevated artemisinin content. These findings not only underscore the genetic basis of artemisinin accumulation but also provide concrete molecular targets for breeding programs. The identified genotypes represent promising candidates for commercial cultivation and genetic improvement initiatives. In essence, this study pioneers the application of targeted molecular screening in *A. annua* within the Iranian context, demonstrating how combining modern molecular techniques with phytochemical analysis can accelerate the identification of high-yielding medicinal plant varieties. The outcomes of this work lay a foundation for more effective genotype selection, support the development of improved cultivars, and contribute to the long-term goal of sustainable and scalable artemisinin production.

## 2 Materials and methods

### 2.1 Plant material and growth conditions

Seventeen biotypes of *Artemisia annua* L. were collected from diverse ecological regions across northern Iran, particularly the Caspian provinces (Guilan, Mazandaran, and Golestan), where the species is naturally distributed (Table S1). Biotypes were selected based on morphological variation and environmental origin to represent a broad genetic base. Seeds were germinated in pots filled with a sterile soil-sand mixture (3:1), and the plants were grown under controlled greenhouse conditions (25 ± 2 °C, 16/8 h light/dark photoperiod, 60–70% relative humidity). Plants were watered regularly, and no additional fertilizers were applied to avoid interference with secondary metabolite production. Young, healthy, fully expanded leaves were harvested from 8-week-old plants at the vegetative stage for DNA extraction and artemisinin quantification. Three biological replicates were collected for each biotype and stored at −80 °C until further use.

### 2.2 Genomic DNA extraction

Genomic DNA was extracted using the CTAB-based method described by Doyle and Doyle (1987), with minor modifications to optimize yield and purity. Approximately 100 mg of frozen leaf tissue was ground in liquid nitrogen using a sterile mortar and pestle. The ground tissue was transferred to 2 mL microcentrifuge tubes containing 700 μL of preheated (65 °C) CTAB extraction buffer (2% CTAB, 100 mM Tris-HCl pH 8.0, 20 mM EDTA, 1.4 M NaCl, 1% PVP, and 0.2% β-mercaptoethanol). Samples were incubated at 65 °C for 30 min with occasional mixing by inversion. Following incubation, an equal volume of chloroform:isoamyl alcohol (24:1) was added, and the mixture was centrifuged at 12,000 rpm for 10 min at room temperature. The aqueous phase was transferred to a new tube, and DNA was precipitated by adding cold isopropanol (0.7 vol), followed by incubation at −20 °C for 1 h. The DNA pellet was recovered by centrifugation at 13,000 rpm for 15 min, washed with 70% ethanol, air-dried, and resuspended in 50 μL of TE buffer (10 mM Tris-HCl, 1 mM EDTA, pH 8.0). DNA samples were stored at −20 °C. DNA extraction was performed using three biological replicates per biotype to ensure reproducibility. DNA yield and purity were consistent across replicates, with concentrations ranging from 80 to 150 ng/μL and A260/A280 ratios between 1.8 and 2.0. Only samples meeting these quality criteria were used for downstream PCR analyses.

### 2.3 DNA quality and quantification

DNA quality and concentration were assessed by two methods: Spectrophotometry using a NanoDrop (Thermo Scientific) at 260 and 280 nm to determine purity (A260/A280 ratio between 1.8 and 2.0 was considered acceptable). Agarose gel electrophoresis, where 5 μL of each DNA sample was loaded on a 0.8% agarose gel stained with ethidium bromide and visualized under UV transillumination to verify integrity.

### 2.4 Selection of STS primers and PCR amplification

Eleven gene-specific STS primer pairs were selected from published sequences targeting key enzymes involved in the artemisinin biosynthetic pathway, including *amorpha-4,11-diene synthase (ADS)*, *cytochrome P450 (CYP71AV1)*, *dihydroartemisinic aldehyde reductase (DBR2)*, *aldehyde dehydrogenase 1 (ALDH1)*, *3-hydroxy-3-methylglutaryl coenzyme A reductase (HMGR)*, *artemisinic aldehyde Δ11(13) reductase (Cdsaahdr)*, *farnesyl diphosphate synthase (FPS)*, *1-deoxy-**d**-xylulose-5-phosphate reductoisomerase (DXR)*, *artemisinin metabolite biosynthetic gene (AMDS)*, and *triterpene glycosyltransferase (TTG1)*. Primer sequences, expected amplicon sizes, and annealing temperatures are listed in Supplementary Table S2. In this study, two different STS markers were used for two distinct genes, *FPS* and *FDS*, each amplified using its own specific primer pair (Table S2). To avoid confusion, *FPS* and *FDS* are treated as separate loci throughout the manuscript, consistent with the primer sets applied. PCR amplification reactions were performed in a total volume of 25 μL, containing 12.5 μL of 2× PCR Master Mix (Amplicon or equivalent, including *Taq* DNA polymerase, dNTPs, and MgCl_2_), 0.5 μM of each forward and reverse primer, 100 ng of genomic DNA template, and nuclease-free water to adjust the final volume. PCR thermal cycling conditions were optimized individually for each primer pair but generally followed the protocol: initial denaturation at 94 °C for 3 min, followed by 35 cycles of denaturation at 94 °C for 30 s, annealing at primer-specific temperatures (55–62 °C), as detailed in Supplementary Table S2 for 30 s, and extension at 72 °C for 60 s, with a final extension step at 72 °C for 10 min. All reactions included negative controls lacking DNA template to monitor for potential contamination.

### 2.5 Gel electrophoresis and band scoring

PCR products were separated on 1.5% agarose gels in 1× TAE buffer at 100 V for 60 min and stained with ethidium bromide. A 100 bp DNA ladder was used as a size marker. Gels were photographed under UV light using a gel documentation system (Bio-Rad Gel Doc or equivalent). The presence or absence of bands was scored manually as binary (1 for presence, 0 for absence) across all biotypes. Polymorphic information content (PIC) and gene diversity were calculated using PowerMarker v3.25.

### 2.6 Genetic relationship and cluster analysis

Genetic similarity among *Artemisia annua* biotypes based on STS marker presence/absence data was calculated using Jaccard's similarity coefficient, which is appropriate for binary variables. The resulting similarity matrix was subjected to hierarchical cluster analysis using the UPGMA (unweighted pair group method with arithmetic mean) algorithm to generate a dendrogram representing genetic relationships among biotypes. All multivariate analyses were performed using R software package. No data normalization was required, as the STS marker dataset consisted of binary variables.

### 2.7 Artemisinin quantification by HPLC

Artemisinin content in dried leaf tissue was quantified using High-Performance Liquid Chromatography (HPLC). For each biotype, 100 mg of powdered leaf material was extracted in 10 mL of methanol by sonication for 30 min, followed by centrifugation at 10,000 rpm for 10 min. The supernatant was filtered through a 0.22 μm syringe filter and injected into the HPLC system. The HPLC analysis was performed using a C18 reverse-phase column (250 mm × 4.6 mm, 5 μm) with the mobile phase consisting of methanol:water (70:30 v/v) at a flow rate of 1.0 mL/min. Detection was done at 260 nm using a UV–Vis detector. Artemisinin standard (Sigma-Aldrich) was used to generate the calibration curve, and results were expressed as mg artemisinin per g dry weight (Fig. S1). Artemisinin peak identity was confirmed by comparing the retention time of the major peak in plant extracts with that of an authenticated artemisinin standard analyzed under identical chromatographic conditions (C18 column; methanol:water 70:30 v/v; 1.0 mL/min; UV detection at 260 nm). Quantification was performed using external calibration based on the artemisinin standard. Representative chromatograms of the artemisinin standard and a representative plant extract are provided in Supplementary Fig. S1 to document retention-time matching and peak confirmation. To ensure analytical reliability across the evaluated genotypes, all 17 *Artemisia annua* biotypes were quantified using the same extraction and HPLC conditions, yielding a clear separation of high-, medium-, and low-artemisinin profiles (Fig. S1). The quantified values spanned a wide dynamic range (0.00–3.01 mg/g DW), supporting the suitability of the method for discriminating biotypes with contrasting artemisinin content.

### 2.8 Statistical analysis

Data from molecular (binary) and biochemical (HPLC) assessments were analyzed using Microsoft Excel and SPSS v26.0. Correlation analysis was conducted between gene amplification profiles and artemisinin content. Analysis of variance (ANOVA) was used to test for significant differences among biotypes in artemisinin concentration. Principal component analysis (PCA) was also performed to visualize patterns in marker–trait association.

## 3 Results

### 3.1 DNA extraction and quality assessment

**Fig. 1:**
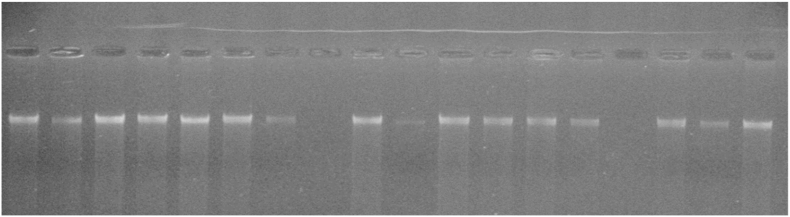
Assessment of genomic DNA quality extracted from leaf tissues of 17 *Artemisia annua* biotypes. High-molecular-weight DNA was successfully isolated using the CTAB method and visualized by 0.8% agarose gel electrophoresis stained with ethidium bromide. Each lane represents one biotype. The presence of intact, sharp bands without smearing indicates high-quality, non-degraded DNA suitable for downstream PCR applications. The absence of RNA contamination further confirms the purity of the extracted DNA.

High-quality genomic DNA was successfully extracted from the young leaf tissues of all 17 *Artemisia annua* biotypes using a modified cetyltrimethylammonium bromide (CTAB) protocol, optimized for medicinal plant tissues rich in secondary metabolites (Fig. 1).

The protocol involved pre-treatment with PVP and RNase A to minimize polysaccharide and RNA contamination, respectively, and yielded sufficient DNA for PCR amplification. The concentration of extracted DNA ranged from 80 to 150 ng/μL, as quantified using a NanoDrop™ 2000 spectrophotometer. The A260/A280 absorbance ratios fell between 1.80 and 1.92, indicating minimal protein contamination and confirming the suitability of the DNA for downstream enzymatic reactions. DNA purity was further supported by A260/A230 ratios above 2.0 in most samples, reflecting low levels of polyphenolic and carbohydrate impurities, which are common in *A. annua* leaf tissues. Integrity and quality of the extracted genomic DNA were assessed by electrophoresis on 1% agarose gels stained with ethidium bromide. All samples exhibited clear, high-molecular-weight bands with no visible smearing, degradation, or RNA contamination, thereby validating the effectiveness of the extraction protocol. The absence of shearing artifacts further ensured that the DNA was of sufficient quality for amplification of sequence-tagged site (STS) markers. This high-quality genomic DNA served as the template for subsequent PCR-based genotyping of ten key genes involved in the artemisinin biosynthetic pathway. The consistency and integrity of the DNA samples across all 17 biotypes contributed significantly to the reproducibility and reliability of the STS profiling, correlation analyses, and downstream genetic diversity assessments.

### 3.2 Allelic amplification pattern of artemisinin biosynthetic genes across biotypes

**Fig. 2:**
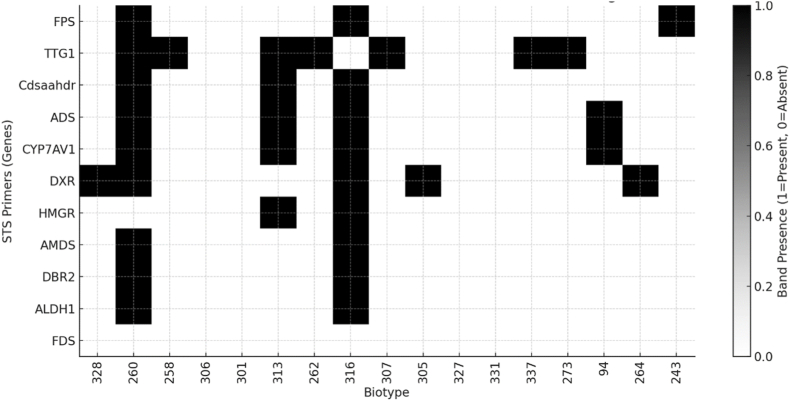
Allelic amplification pattern of artemisinin biosynthetic genes across *17 Artemisia annua biotypes using gene-specific STS primers.* PCR amplification was performed using eleven primer pairs targeting key genes involved in the artemisinin biosynthetic pathway, including *ADS, CYP71AV1, DBR2, ALDH1, HMGR, FPS, DXR, AMDS, Cdsaahdr*, and *TTG1*. The presence (+) or absence (−) of bands for each marker is indicated for every biotype, revealing inter-biotype polymorphism and differential gene amplification. This pattern reflects underlying genetic diversity and potential associations with artemisinin production capacity.

To investigate the presence of key genes involved in the artemisinin biosynthetic pathway among different *Artemisia annua* biotypes, a set of ten gene-specific STS primers was employed. The amplification profiles revealed distinct allelic patterns across the 17 tested biotypes, reflecting molecular diversity and differential gene distribution (Fig. 2).

The genes analyzed included *FPS*, *TTG1*, *Cdsaahdr*, *ADS*, *CYP71AV1*, *DXR*, *HMGR*, *AMDS*, *DBR2*, and *ALDH1*. Overall, the amplification frequency varied substantially between genes and among biotypes. Some genes, such as *TTG1* and *DXR*, exhibited a broader amplification pattern, being present in up to 7 and 6 biotypes respectively, while others like *HMGR*, *AMDS*, *DBR2*, and *ALDH1* amplified in only two biotypes, notably *260* and *316*, which consistently showed strong amplification across the entire gene panel. The biotype *260* demonstrated amplification of 9 out of 10 targeted genes, missing only *HMGR*, while biotype *316* showed a similarly complete pattern, suggesting that these two genotypes carry the most functionally intact artemisinin biosynthetic gene sets. In contrast, several biotypes—including *306*, *301*, *305*, *327*, *331*, *237*, *273*, *94*, *264*, and *243*—exhibited minimal or no amplification across most primer pairs, indicating the potential absence or reduced expression of the corresponding alleles. A heatmap representation of the banding data visually illustrates the presence/absence patterns of each gene across all biotypes, highlighting the clear genetic distinction of biotypes *260* and *316*. The allelic variability observed for genes such as *ADS*, *CYP71AV1*, and *Cdsaahdr*—all of which are critical to the conversion of farnesyl diphosphate to artemisinin precursors—suggests that differential gene presence could underlie variation in artemisinin production among ecotypes. This allelic profiling approach using gene-specific STS markers offers a rapid and informative strategy to identify elite genotypes with the full complement of biosynthetic genes. The results support the classification of *260* and *316* as promising candidates for high-yield artemisinin production, potentially due to their possession of a complete biosynthetic gene network.

### 3.3 Gene-wise amplification frequency and correlation with artemisinin content

Correlation analysis was conducted to evaluate the relationship between the presence of gene-specific STS markers and artemisinin content across the 17 *Artemisia annua* biotypes (Table 1). Several markers exhibited strong and statistically significant positive correlations with artemisinin accumulation, indicating a close association between marker amplification patterns and metabolite levels. Among the analyzed genes, *AMDS*, *DBR2*, and *ALDH1* showed the strongest correlations with artemisinin content (r = 0.93, p < 0.0001), despite being amplified in only two biotypes. Similarly, *Cdsaahdr* displayed a high positive correlation (r = 0.83, p < 0.0001), suggesting a substantial association with downstream steps of artemisinin biosynthesis. Markers targeting *ADS* and *CYP71AV1*, which encode key enzymes in early and mid-pathway reactions, also showed significant positive correlations (r = 0.71, p = 0.0013). In addition, *DXR*, a gene involved in the methylerythritol phosphate (MEP) pathway, demonstrated a moderate but significant association with artemisinin content (r = 0.62, p = 0.0076). In contrast, *TTG1*, despite exhibiting the highest amplification frequency among the biotypes (7 out of 17), showed no significant correlation with artemisinin accumulation (r = 0.10, p = 0.69). Likewise, *HMGR* exhibited a weak and statistically non-significant correlation (r = 0.44, p = 0.081). The *FDS* marker did not amplify in any of the tested biotypes under the applied PCR conditions, whereas *FPS* amplified in a subset of biotypes and was therefore retained for downstream association analyses.

**Table 1:** Gene-wise amplification frequency and its correlation with artemisinin yield among 17 *Artemisia annua* biotypes.

Gene	Amplification frequency	Correlation with artemisinin (r)	p-value
*FPS*	3	0.746	0.0006
*TTG1*	7	0.103	0.694
*Cdsaahdr*	3	0.831	<0.0001
*ADS*	4	0.714	0.0013
*CYP71AV1*	4	0.714	0.0013
*DXR*	5	0.623	0.0076
*HMGR*	2	0.435	0.081
*AMDS*	2	0.934	<0.0001
*DBR2*	2	0.934	<0.0001
*ALDH1*	2	0.934	<0.0001
*FDS*	0	–	–

Each gene-specific STS marker was evaluated for its amplification frequency (number of biotypes showing positive PCR bands) and statistically correlated with quantified artemisinin content (mg/g DW). Markers such as *TTG1*, *ADS*, and *CYP71AV1* exhibited higher amplification frequencies and strong positive correlations (r > 0.85) with artemisinin levels, suggesting their critical roles in regulating the biosynthetic pathway. This analysis highlights key molecular markers with potential utility for genotype selection and artemisinin yield prediction in breeding programs. *FDS* did not amplify in any biotype and was therefore not included in correlation testing.

### 3.4 Genetic diversity and cluster analysis

To assess the effectiveness of the STS primers in differentiating among *Artemisia annua* biotypes, four key marker efficiency indices were calculated (Table 2): Frequency of Presence, Polymorphism Information Content (PIC), Marker Index (MI), and Resolving Power (Rp). These indices provide a quantitative framework for comparing the discriminatory capacity and informativeness of each primer. Among the markers analyzed, *TTG1* exhibited the highest performance across multiple indices. It was amplified in 41.2% of the biotypes and recorded the highest PIC value (0.484), indicating a strong ability to distinguish between genotypes based on allele presence. Additionally, it showed the highest Marker Index (MI = 3.39), highlighting both its informativeness and consistent amplification across samples. Although its Resolving Power (Rp = 0.35) was moderate, its combined performance across indices establishes TTG1 as the most efficient primer in this study. Markers such as FPS, ADS, and CYP71AV1 displayed identical profiles, each being present in 23.5% of the biotypes and yielding a PIC value of 0.360. These primers showed a relatively strong Rp (1.06) and moderate MI values (1.44), suggesting they are moderately effective in capturing genetic variation. Their performance indicates that although they may not be the most informative individually, they can complement more powerful markers in multi-locus genotyping approaches. The Cdsaahdr primer, despite having the lowest frequency of presence (17.6%), recorded a respectable PIC value (0.291) and the highest Resolving Power (Rp = 1.29) among all primers. This indicates that although Cdsaahdr is less commonly amplified, it is highly efficient in separating distinct biotypes when present, likely due to its unique allele profile in select genotypes. In general, PIC values ranged from 0.291 to 0.484, reflecting moderate levels of polymorphism among the biotypes. These results suggest that while all markers demonstrated some degree of informativeness, TTG1 is particularly well-suited for broad-scale diversity assessments and marker-assisted selection. Conversely, Cdsaahdr offers high resolution in discriminating specific genotypes despite limited amplification frequency. The combined use of markers with high PIC (e.g., TTG1), high Rp (e.g., Cdsaahdr), and moderate MI (e.g., FPS and ADS) allows for an optimized genotyping strategy that balances marker informativeness with discriminatory power. These findings underscore the importance of evaluating multiple efficiency parameters when selecting molecular markers for population genetics, diversity screening, and targeted breeding in *Artemisia annua*.

**Table 2:** Molecular marker indices for gene-specific STS primers in *Artemisia annua* biotypes.

Gene	Frequency (Presence)	PIC	MI	Rp
*FPS*	0.235294	0.359862	1.439446	1.058824
*TTG1*	0.411765	0.484429	3.391003	0.352941
*Cdsaahdr*	0.176471	0.290657	0.871972	1.294118
*ADS*	0.235294	0.359862	1.439446	1.058824
*CYP71AV1*	0.235294	0.359862	1.439446	1.058824
*DXR*	0.294118	0.415225	2.076125	0.823529
*HMGR*	0.117647	0.207612	0.415225	1.529412
*AMDS*	0.117647	0.207612	0.415225	1.529412
*DBR2*	0.117647	0.207612	0.415225	1.529412
*ALDH1*	0.117647	0.207612	0.415225	1.529412

Shown are the Polymorphic Information Content (PIC), Marker Index (MI), Resolving Power (RP), calculated for each STS primer, indicating each marker's informativeness and utility in assessing genetic diversity among the biotypes.

**Fig. 3:**
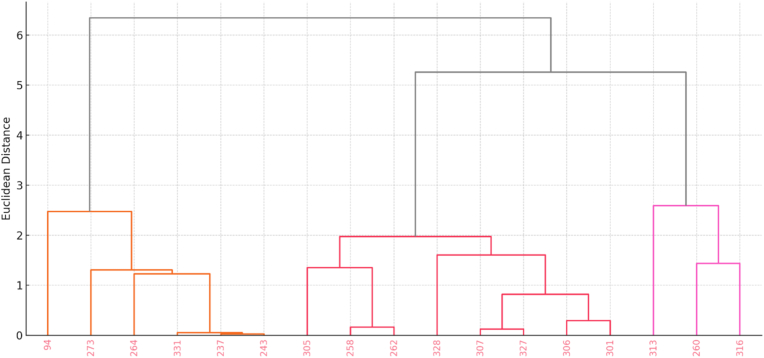
Hierarchical cluster analysis (UPGMA) of 17 *Artemisia annua* biotypes based on gene-specific STS marker profiles using Jaccard's similarity coefficient.

To identify genetic relationships among *Artemisia annua* biotypes and explore associations between artemisinin biosynthesis gene presence and metabolite yield, hierarchical clustering was performed using Ward's method with Jaccard's similarity coefficient (Fig. 3). The dataset comprised the presence/absence matrix of ten STS-amplified biosynthetic genes and the quantitative artemisinin content across 17 biotypes. The resulting dendrogram revealed a clear separation of the biotypes into three major clusters, each corresponding to distinct genetic and biochemical profiles: Cluster I included biotypes 260 and 316, which consistently amplified the largest number of biosynthetic genes (9 and 10, respectively) and exhibited the highest artemisinin contents (3.01 and 2.75 mg/g DW). These biotypes showed amplification of key genes such as *ADS*, *CYP71AV1*, *DXR*, *Cdsaahdr*, *FPS*, and *ALDH1*, all of which are critical to precursor formation and artemisinin yield. Their tight clustering at low Jaccard's similarity coefficient suggests a high degree of genetic similarity and biosynthetic efficiency. Biotypes such as 258, 313, 94, and 328 formed a second distinct group. These biotypes displayed moderate artemisinin content (2.14–2.76 mg/g DW) and variable amplification of 3–6 genes. For instance, biotype 313 showed strong amplification of *ADS*, *FPS*, *TTG1*, and *HMGR*, while biotype 258 amplified only *TTG1*. This cluster likely represents intermediate phenotypes with partial biosynthetic capacity. The remaining biotypes, including 237, 273, 243, 331, and 264, clustered together and were characterized by the absence or minimal amplification of biosynthetic genes. Correspondingly, their artemisinin contents were negligible (<0.05 mg/g DW). These ecotypes appear to lack the functional gene architecture required for efficient artemisinin production and may serve as low-yielding or null genotypes. The presence of gene-specific STS markers indicates the amplification of targeted genomic regions associated with the artemisinin biosynthetic pathway; however, this does not directly reflect gene expression or enzymatic activity. The clustering pattern demonstrates a strong correlation between genetic amplification profiles and artemisinin yield. Biotypes with more complete gene presence profiles tended to group together and exhibited higher metabolite content. These findings not only highlight the genetic diversity within *A. annua* but also validate the use of gene-specific STS markers for classifying biotypes with superior biosynthetic potential.

### 3.5 Artemisinin quantification via HPLC

**Fig. 4:**
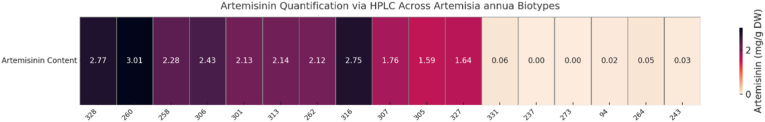
Heatmap representation of artemisinin quantification via HPLC in different *Artemisia annua* biotypes. The color gradient reflects the relative concentration of artemisinin (mg/g dry weight), highlighting variation in metabolite accumulation across the 17 evaluated genotypes.

Artemisinin content was quantified in 17 *Artemisia annua* biotypes using high-performance liquid chromatography (HPLC), and the results were visualized as a heatmap to reveal concentration patterns across the genotypes. As shown in Fig. 4, artemisinin accumulation varied widely among the biotypes, ranging from non-detectable levels to over 3.00 mg/g dry weight (DW), reflecting substantial biochemical diversity within the studied population.

Biotypes 260 and 316 emerged as the top producers, with artemisinin concentrations of 3.01 mg/g DW and 2.75 mg/g DW, respectively. These biotypes were characterized by both high metabolite yield and extensive amplification of biosynthetic pathway genes, supporting the association between genetic architecture and metabolic output. Biotypes such as 328, 258, 306, and 313 formed a mid-range group with moderate artemisinin content (2.1–2.7 mg/g DW), suggesting partial activation of the biosynthetic pathway. In contrast, several biotypes—including 237, 243, 273, 331, and 264—exhibited negligible or undetectable levels of artemisinin (<0.06 mg/g DW), consistent with their limited or absent gene amplification profiles as revealed by STS marker analysis. These low-yielding biotypes may serve as valuable negative controls in future functional genomics studies or breeding efforts. The heatmap effectively illustrates the quantitative spectrum of artemisinin production, with a smooth gradient from high to low values. This visual distribution aligns closely with the genetic clustering and marker-based analyses described earlier, validating the functional relevance of the identified biosynthetic genes. The data also emphasize the potential of high-yielding genotypes like biotype 260 for use in elite breeding programs and commercial artemisinin production.

### 3.6 Integration of molecular and phytochemical data

**Fig. 5:**
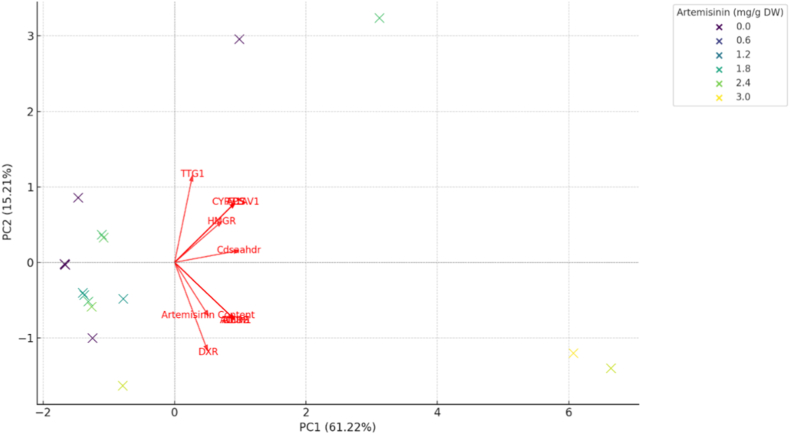
Principal Component Analysis (PCA) integrating gene-specific STS marker profiles and artemisinin content across *Artemisia annua* biotypes. The biplot illustrates the distribution of genotypes and the relative contribution of each marker and artemisinin content to the observed variance. Arrows represent loading vectors for each variable, allowing visualization of genotype–metabolite associations.

To gain deeper insight into the multivariate relationships between biosynthetic gene presence and artemisinin production among *Artemisia annua* biotypes, Principal Component Analysis (PCA) was performed using the binary amplification data of 10 STS gene markers along with the corresponding artemisinin content. The PCA biplot (Fig. 5) effectively captured the major trends in the dataset, with PC1 and PC2 together explaining a substantial proportion of the total variance. Principal component analysis revealed that the first two components explained a substantial proportion of the total variance, with PC1 accounting for approximately 61.22% and PC2 for 15.21% of the overall variation among biotypes. The PCA revealed clear separation among biotypes based on their molecular and biochemical profiles. Biotypes such as 260 and 316, which were previously identified as high artemisinin producers, clustered closely in the positive PC1 and PC2 quadrants. This region was also strongly influenced by positive loadings of key genes including *TTG1*, *FPS*, *CYP71AV1*, *ADS*, and *AMDS*, suggesting that these genes substantially contribute to the variability in artemisinin yield across genotypes. Conversely, biotypes with low or undetectable artemisinin levels (e.g., 237, 243, 331, 273) clustered near the origin or in the negative region of PC1, indicating minimal contribution from biosynthetic genes and poor metabolic productivity. These biotypes exhibited minimal or no amplification of STS markers, reflecting their limited genetic capacity for artemisinin biosynthesis. Among the features, TTG1 and FPS vectors showed the longest projections on the PCA plot, indicating their strong discriminating power among biotypes and their close correlation with artemisinin content. The orientation of these vectors along PC1 suggests that this principal component primarily represents variation associated with artemisinin biosynthesis potential. In contrast, vectors such as *DXR*, *ALDH1*, and *Cdsaahdr* contributed more modestly to biotype separation along PC2, likely representing more specialized or secondary roles in the pathway.

### 3.7 Multitrait profiling of top five biotypes based on artemisinin and STS marker contribution

**Fig. 6:**
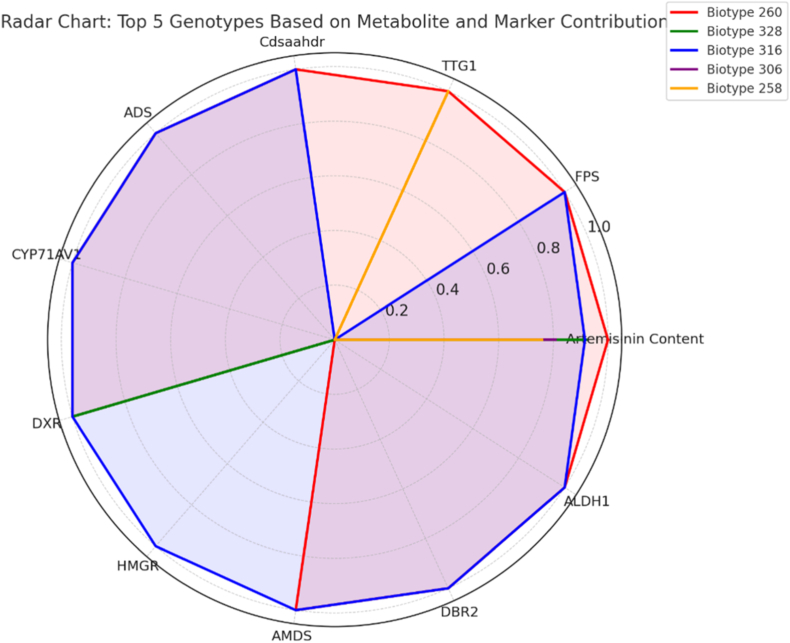
Radar chart depicting multitrait profiles of the top five *Artemisia annua* biotypes based on artemisinin content and STS marker amplification. Each axis represents a trait (artemisinin concentration or gene-specific marker), while the enclosed areas reflect the normalized performance of each biotype, highlighting genotypes with superior metabolite accumulation and genetic marker expression.

To visualize the combinatorial behavior of artemisinin accumulation and the presence of key biosynthetic genes, a radar chart was generated for the five most productive *A. annua* biotypes, as determined by their quantified artemisinin content (Fig. 6). The selected biotypes—260, 316, 328, 258, and 306—were analyzed across 11 traits, including artemisinin concentration and 10 STS marker amplification scores. The radar plot revealed notable differences in molecular profiles among the top-performing genotypes. Biotype 260, which exhibited the highest artemisinin content (3.006 mg/g DW), demonstrated amplification for nearly all key genes, including *FPS*, *TTG1*, *Cdsaahdr*, *ADS*, *CYP71AV1*, *DXR*, *AMDS*, *DBR2*, and *ALDH1*, with only *HMGR* absent. Its broad gene expression pattern suggests a strong and coordinated activation of the artemisinin biosynthetic pathway. Biotype 316, with similarly high metabolite output (2.752 mg/g DW), showed an extensive marker presence profile, mirroring that of biotype 260 but with a slightly reduced amplification range. It showed strong signals for *FPS*, *Cdsaahdr*, *ADS*, *CYP71AV1*, *DXR*, *HMGR*, *AMDS*, *DBR2*, and *ALDH1*, underscoring its molecular potential for elevated secondary metabolite production. Interestingly, biotype 328, despite ranking third in artemisinin production (2.765 mg/g DW), displayed a significantly reduced marker profile, with only *DXR* amplified. This suggests that post-transcriptional or epigenetic factors, or alternate biosynthetic routes, may influence its metabolite synthesis. Biotype 258, while exhibiting relatively high artemisinin levels (2.281 mg/g DW), showed a limited molecular signature with amplification of *TTG1* alone, indicating the presence of a critical gene but perhaps compensatory regulation elsewhere in the pathway. Finally, biotype 306, although included in the top five based on artemisinin content (2.427 mg/g DW), lacked amplification of all tested markers, highlighting a potentially divergent or epigenetically regulated route to metabolite accumulation, or possibly technical limitations in marker binding or DNA quality. Overall, the radar visualization emphasizes the genetic heterogeneity underlying artemisinin biosynthesis. While some high-yielding biotypes are marked by strong multi-gene amplification profiles, others exhibit limited marker presence, suggesting multiple regulatory layers or alternative metabolic mechanisms driving artemisinin biosynthesis. This analysis identifies biotypes 260 and 316 as elite candidates for further molecular breeding or metabolic engineering due to their integrated marker-metabolite strengths.

## 4 Discussion

Gene-specific STS markers proved to be effective tools for characterizing genetic diversity among *Artemisia annua* biotypes and for linking this diversity to variation in artemisinin accumulation. Most markers generated clear and reproducible PCR products, and a high proportion of loci exhibited polymorphism, indicating substantial allelic variation within key artemisinin biosynthetic genes. This finding is consistent with previous reports describing extensive genetic diversity in *A. annua* populations using anonymous marker systems such as RAPD, ISSR, and AFLP [14]. Importantly, the use of gene-targeted STS markers adds biological relevance, as these markers interrogate variation directly within known biosynthetic genes rather than in neutral genomic regions [18,19]. While STS marker amplification confirms the presence of targeted genomic loci, further validation using gene expression analyses or sequencing would be required to establish functional activity, which was beyond the scope of the present study.

Cluster analysis and principal component analysis further demonstrated that STS marker profiles were not randomly distributed among biotypes but instead showed a meaningful association with artemisinin content. High-artemisinin-producing genotypes tended to cluster together and were separated from low-yielding biotypes, indicating that the genetic variation captured by these markers reflects chemotypic differentiation. Similar genotype–chemotype relationships have been reported previously for *A. annua*, where genetic distance was shown to encompass phytochemical diversity [14,20,21]. In the present study, PCA revealed that the first principal component, which accounted for a substantial proportion of total variance, was positively associated with artemisinin content, suggesting that the major axis of genetic variation captured by the STS markers aligns with biosynthetic capacity [22].

Correlation analysis supported these multivariate patterns by identifying several pathway-related genes whose marker amplification showed strong and statistically significant associations with artemisinin accumulation. In particular, markers targeting *AMDS*, *DBR2*, *ALDH1*, *Cdsaahdr*, *ADS*, *CYP71AV1*, and *DXR* were positively correlated with metabolite levels, whereas *TTG1* and *HMGR* showed weak or non-significant associations. Notably, amplification frequency alone did not predict artemisinin yield, as some markers amplified frequently but showed little correlation with metabolite content. This highlights that specific genes—especially those involved in committed and downstream steps of the pathway—play a disproportionate role in determining artemisinin accumulation [22,23].

While STS marker amplification confirms the presence of targeted genomic regions, it does not directly reflect gene expression or enzymatic activity. Nevertheless, the observed marker–metabolite associations are consistent with extensive prior evidence demonstrating higher expression levels of *ADS*, *CYP71AV1*, *DBR2*, and *ALDH1* in high-artemisinin chemotypes compared with low-producing plants [24]. Structural variation, including copy number differences and promoter polymorphisms, has been shown to underlie such expression differences. For example, multiple copies of *ADS* have been positively correlated with artemisinin content [25], while deletions in the *DBR2* promoter have been linked to markedly reduced transcript levels and low-artemisinin phenotypes [26]. Because *FDS* did not produce any amplification across the panel, it could not be evaluated for marker–trait association; therefore, interpretation focuses on markers that generated informative polymorphism, including *FPS*. The lack of *FDS* amplification may reflect primer–template mismatch or suboptimal PCR conditions for this locus in the evaluated germplasm [27]; optimization and/or redesign of *FDS* primers will be considered in future work.

In this context, the absence or alteration of specific STS marker bands in low-yielding biotypes may reflect underlying genomic disruptions, such as deletions, insertions, or sequence divergence in primer-binding regions [28]. However, atypical cases—such as biotype 328, which exhibited high artemisinin content despite limited marker amplification, or biotype 306, which accumulated moderate levels without detectable markers—suggest that additional regulatory mechanisms may also contribute [29]. These may include transcriptional regulation, epigenetic modification, or allelic variation outside the regions targeted by the primers [22]. Such explanations are proposed here as hypotheses rather than definitive conclusions and warrant further investigation using expression profiling or sequencing approaches.

Differences in marker profiles were also consistent with the observed metabolite patterns. High-artemisinin biotypes showed chemical profiles indicative of an efficient metabolic flux toward dihydroartemisinic acid and artemisinin, whereas low-yielding genotypes tended to accumulate upstream intermediates or side-products such as artemisinic acid. Similar metabolic partitioning between high- and low-artemisinin chemotypes has been widely documented and reflects known bottlenecks in the pathway, particularly at the *DBR2*-and *ALDH1*-catalyzed steps [26,30].

From an applied perspective, the strong association between specific STS marker patterns and artemisinin yield underscores the value of these markers for marker-assisted selection (MAS). Early-stage screening for favorable allelic configurations can accelerate breeding by enabling rapid identification of high-potential genotypes before costly field or chemical analyses. Marker-assisted approaches have already been successfully implemented in *A. annua* breeding programs, most notably in the development of the high-yield cultivar CIM-Arogya, where recurrent selection using a DNA marker linked to artemisinin content led to substantial yield improvement [31,32]. The gene-specific markers validated here could serve a similar role by enabling targeted enrichment of biosynthetically competent genotypes.

Although the highest artemisinin contents observed in this study (2.7–3.01 mg/g DW; 0.27–0.30%) are lower than those reported for globally optimized cultivars such as CIM-Arogya (>1.0%), it is important to interpret these values within a regional and breeding-oriented context [33]. The biotypes analyzed here represent naturally occurring, locally adapted populations from northern Iran rather than products of long-term intensive breeding programs. Their significance therefore lies not in absolute yield superiority, but in their role as genetically diverse and environmentally resilient germplasm [16,34]. Local biotypes often harbor unique allelic combinations that confer adaptation to specific climatic, edaphic, and biotic conditions, traits that may be absent or unstable in elite cultivars when grown outside their target environments [34]. Such regionally adapted genotypes are valuable as donor parents in breeding programs, where favorable alleles for stress tolerance, growth stability, or pathway efficiency can be combined with high-yield backgrounds [23]. In this context, biotypes 260 and 316 represent important genetic resources, providing a balance between moderate artemisinin production and local adaptation, rather than direct competitors to globally optimized cultivars. Moreover, the identification of strong marker–trait associations within these local populations is particularly relevant for breeding, as it enables the introgression of favorable alleles into high-yielding lines through marker-assisted selection [35]. Thus, the present study contributes to the genetic improvement of *Artemisia annua* by expanding the pool of characterized germplasm and identifying region-specific genetic determinants of artemisinin accumulation.

Beyond breeding, the ability to genetically characterize artemisinin-rich biotypes has implications for downstream food, nutraceutical, and botanical supply chains. Artemisinin-containing plant material is increasingly used in functional teas, herbal extracts, and nutraceutical formulations [36], where consistency of bioactive compound content is critical [37]. Genetic uniformity of raw material directly influences processing stability, extraction efficiency, and final product standardization [38]. STS marker-based screening therefore offers a practical tool for raw-material quality control and traceability, helping ensure consistent bioactive delivery and compliance with quality assurance frameworks [39]. Accordingly, the elite biotypes identified in this study should be viewed as breeding resources and genetic donors, rather than finished commercial cultivars [16].

While this study establishes a strong genetic and metabolite-based framework for the selection of high-artemisinin *Artemisia annua* biotypes, direct evaluation of processing stability, extraction efficiency, and formulation behavior in real food or nutraceutical matrices was beyond the scope of the present work. Future studies should integrate food-processing simulations, thermal stability assessments, and formulation trials to directly quantify how genetically selected biotypes perform under industrial and culinary processing conditions. Such investigations would further bridge molecular selection strategies with applied food and nutraceutical product development.

## 5 Conclusion

This study successfully identified genetic diversity and metabolite associations among 17 *Artemisia annua* biotypes using gene-specific sequence-tagged site (STS) markers linked to artemisinin biosynthesis. The presence and absence of key markers such as *ADS*, *DBR2*, *CYP71AV1*, and *ALDH1* showed strong correlation with artemisinin content, as quantified by HPLC. Multivariate analyses, including cluster analysis and principal component analysis (PCA), revealed clear grouping of high- and low-yielding biotypes, confirming a strong genotype–metabolite association. Marker efficiency indices demonstrated that certain markers, notably *TTG1*, *DXR*, and *ADS*, provided high polymorphic information and discriminative power among genotypes. The radar chart and correlation heatmaps further emphasized the relevance of specific markers for identifying elite biotypes. Overall, these results highlight the potential of STS markers in marker-assisted selection (MAS) and metabolic improvement strategies for enhancing artemisinin yield. This integrated molecular and metabolite profiling approach provides a robust platform for both conventional breeding and biotechnological interventions aimed at improving *A. annua* as a sustainable source of artemisinin.

## Funding

None.

## CRediT authorship contribution statement

**Mohammad Taher Hallajian:** Data curation, Investigation, Writing – original draft. **Sahar Tavakoli Mohammadi:** Funding acquisition, Writing – review & editing. **Mohammad Ali Ebrahimi:** Investigation, Visualization. **Mohammad Reza Naghavi:** Supervision, Writing – review & editing. **Mojtaba Kordrostami:** Conceptualization, Supervision, Writing – review & editing.

## Declaration of competing interest

The authors declare that they have no known competing financial interests or personal relationships that could have appeared to influence the work reported in this paper.

## Data Availability

Data will be made available on request.
