# Supplementary material for: Characterization of high-artemisinin yielding Artemisia annua bioecotypes using gene-specific STS markers and HPLC for quality-oriented selection
Source: Biochem Biophys Rep. 2026 May 23;46:102637. doi: 10.1016/j.bbrep.2026.102637 (PMC13224030; doi:10.1016/j.bbrep.2026.102637)
Supplement: Multimedia component 1 [file mmc1.docx]

**Table S1.** Geographic origin and collection details of Artemisia annua biotypes used in this study.

| **Biotype ID** | **Province** | **Collection locality (Iran)** | **Latitude (N)*** | **Longitude (E)*** |
| --- | --- | --- | --- | --- |
| 328 | Mazandaran | 20 km after Galugah | 36.74 | 53.85 |
| 260 | Gilan | Kharma village | 37.23 | 49.92 |
| 258 | Gilan | Between Lahijan and Amlash | 37.10 | 50.10 |
| 306 | Mazandaran | Qaemshahr toward Sari | 36.46 | 52.90 |
| 301 | Mazandaran | Babol–Babolsar | 36.55 | 52.68 |
| 313 | Mazandaran | Takam | 36.60 | 53.05 |
| 262 | Gilan | Between Rahimabad and Garmabad | 36.80 | 50.30 |
| 316 | Mazandaran | Sari | 36.56 | 53.06 |
| 307 | Mazandaran | 68 km from Kiasar | 36.35 | 53.75 |
| 94 | East Azerbaijan | Azerbaijan region | 38.07 | 46.30 |
| 264 | Gilan | Rudsar | 37.14 | 50.29 |
| 243 | Gilan | Talesh toward Astara | 38.30 | 48.87 |
| 305 | Mazandaran | Behmiz toward Sari | 36.48 | 53.00 |
| 327 | Mazandaran | 7 km toward Galugah | 36.73 | 53.82 |
| 331 | Mazandaran | Qaemshahr toward Firuzkuh | 36.42 | 52.80 |
| 237 | Gilan | 10 km from Masuleh | 37.15 | 48.98 |
| 273 | Mazandaran | Ramsar | 36.92 | 50.65 |
