# Supplementary material for: Characterization of high-artemisinin yielding Artemisia annua bioecotypes using gene-specific STS markers and HPLC for quality-oriented selection
Source: Biochem Biophys Rep. 2026 May 23;46:102637. doi: 10.1016/j.bbrep.2026.102637 (PMC13224030; doi:10.1016/j.bbrep.2026.102637)
Supplement: Multimedia component 2 [file mmc2.docx]

| **Primer** | **Sequence** | **Annealing Temperature (°C)** | **Primer** | **Annealing Temperature (°C)** | **Sequence** |
| --- | --- | --- | --- | --- | --- |
| **DXR-R** | **5'-AGAACAAAGGGACCGCCAGC-3'** | **60** | **Cdsaahdr-R** | **58** | **5'- CCGTCGACCTACACCAATTGCAGGGC-3'** |
| **DXR -F** | **5'-ACGGGATTCAATGTTCGGCTG-3'** |  | **FDS - F** | **57** | **5'- TATTCACCG CCG AATTGTTC -3'** |
| **FPS-R** | **5'-GACTGCTTTGCTTGGGCGATT-3'** | **60** | **FDS-R** |  | **5'- AAGGAT TTCAACACCGCTTG -3'** |
| **FPS-F** | **5'-TTTTGGTGCTCCCGAGGTGA-3'** |  | **AMDS-F** | **60** | **5'- CACAAGGAAGAGCTCAGCCATGTGTG -3'** |
| **ADS-R** | **5'-AGTGCCCGTTGTATTTCG-3'** | **62** | **AMDS-R** |  | **5'-TCATTTAGGCGTCGACCAAGTATACCT-3'** |
| **ADS-F** | **5'-TATGGTTCCGTCTTATGC-3'** |  | **HMGR-F** | **61** | **5'- TTGTGTGCGAGGCAGTAAT -3'** |
| **TTG1-F** | **5'-CCAGCTTGATGACACCAACGG-3'** | **61** | **HMGR-R** |  | **5'- CCTGACCAGTGGCTATAAAGA-3'** |
| **TTG1-R** | **5'- CCCACTCCCAGCCCCACTAC-3'** |  | **DBR2-F** | **62** | **5'-GGTGGGTCATTAGAAAACCGCTG -3'** |
| **CYP71AV1-F** | **5'- TTCGTTTACAAGTTCGCTAC-3'** | **62** | **DBR2-R** |  | **5'- CCTGACCAGTGGCTATAAAGA -3'** |
| **CYP71AV1-R** | **5'- TTCGGAGATGACACCACA-3'** |  | **ALDH1-F** | **60** | **5'-CACAAGGAAGAGCTCAGCCATGTGTG -3'** |
| **Cdsaahdr-F** | **5'- CCGGATCCATGGCGTCTTTGCAGCTAAC-3'** | **58** | **ALDH1-R** |  | **5'-TCATTTAGGCGTCGACCA AGTATACCT -3'** |

**Table S1**. Primer sequence of the studied markers
