# Supplementary material for: Characterization of high-artemisinin yielding Artemisia annua bioecotypes using gene-specific STS markers and HPLC for quality-oriented selection
Source: Biochem Biophys Rep. 2026 May 23;46:102637. doi: 10.1016/j.bbrep.2026.102637 (PMC13224030; doi:10.1016/j.bbrep.2026.102637)
Supplement: Multimedia component 3 [file mmc3.docx]

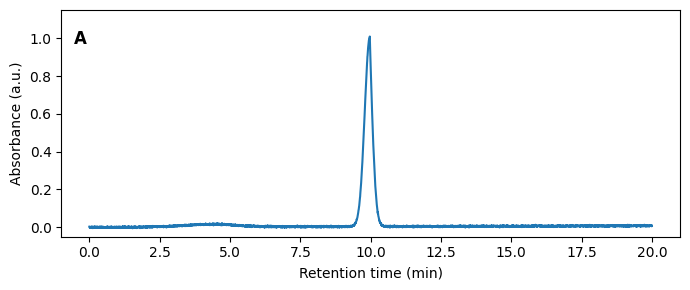


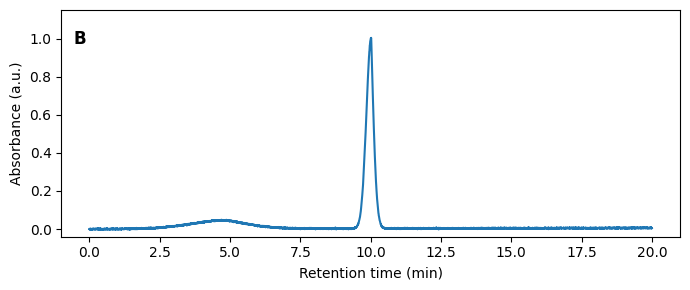


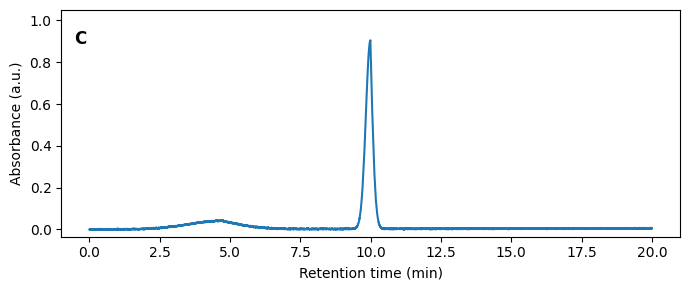


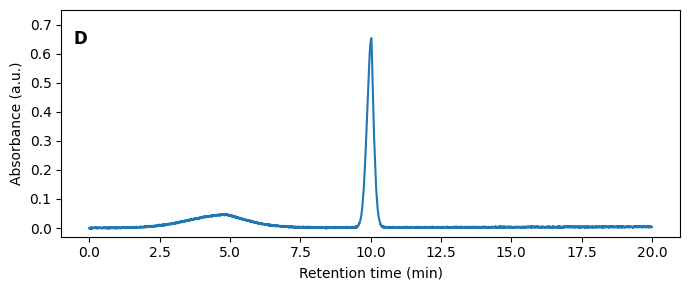


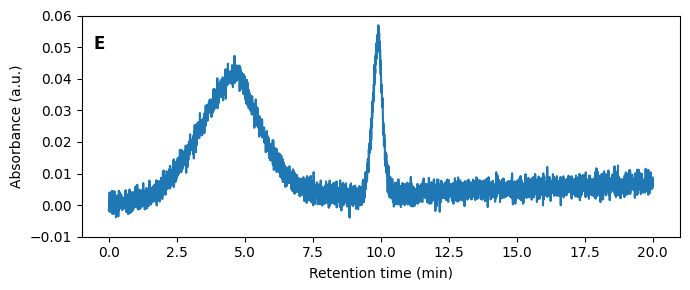


**Figure S1.** HPLC chromatograms demonstrating artemisinin peak identity and relative abundance.
(A) Artemisinin standard chromatogram and representative chromatograms of *Artemisia annua* leaf extracts from (B) biotype 260 (high artemisinin content), (C) biotype 316 (high artemisinin content), (D) biotype 306 (moderate artemisinin content), and (E) biotype 331 (low or trace artemisinin content), recorded at 260 nm. The artemisinin peak appears at a consistent retention time across all panels, while variations in peak intensity reflect differences in relative artemisinin abundance among biotypes.
